# Autoimmune Gastro-Pancreatitis with Anti-Protein Disulfide Isomerase-Associated 2 Autoantibody in Aire-Deficient BALB/cAnN Mice

**DOI:** 10.1371/journal.pone.0073862

**Published:** 2013-08-26

**Authors:** Hironori Kurisaki, Yukihiro Nagao, Seiho Nagafuchi, Masao Mitsuyama

**Affiliations:** 1 Department of Microbiology, Kyoto University Graduate School of Medicine, Kyoto, Japan; 2 Department of Medical Science and Technology, Graduate School of Medical Sciences, Kyushu University, Fukuoka, Japan; Université Paris Descartes, France

## Abstract

Although the autoimmune regulator (Aire) knockout (KO) mouse model has been reported to present various organ-specific autoimmune diseases depending on genetic background, autoimmune pancreatitis in mice of BALB/c background has not yet been reported. Here, we report that Aire KO mice with BALB/cAnN background showed significant lymphoid cell infiltration in the pancreas and stomach. To examine whether the phenotype in the pancreas and stomach is due to autoimmune reaction associated with autoantibody production, indirect immunofluorescence staining followed by Western blot analysis was performed. Consequently, the autoantibody against pancreas and stomach was detected in the sera of Aire KO mice, and the target antigen of the autoantibody was identified as protein disulfide isomerase-associated 2 (Pdia2), which was reported to be expressed preferentially in the pancreas and stomach. Thus, Aire KO mice of BALB/cAnN background can serve as a useful animal model for autoimmune gastro-pancreatitis with anti-Pdia2 autoantibody production.

## Introduction

Autoimmunity is caused by failure of immunological tolerance for self-tissue antigen. Self-reactive immune cells are normally deleted due to so-called self-tolerance system. The self-tolerance develops mainly in central and also in peripheral lymphoid tissues. A negative selection to remove auto-reactive T cells is mainly accomplished in thymic medulla [1], [2]. However, in thymic medullary epithelial cells (mTECs), expression of a number of ectopic genes encoding tissue-specific peripheral antigens (TSAs) has been observed. When the self-tolerance breaks down due to some causes, an immune response to the self-antigen is induced, resulting in the development of autoimmune diseases.

Autoimmune polyendocrinopathy-candidiasis ectodermal dystrophy (APECED), also known as autoimmune polyendocrine syndrome type 1 (APS-1), is an autoimmune disease characterized by chronic mucocutaneous candidiasis and the autoimmune disease of unique organs [3], [4]. APECED patients develop candidiasis in childhood, and then polyendocrine insufficiency including Addison's disease and hypoparathyroidism. In addition to these three major symptoms, various clinical manifestations including type 1 diabetes, hypogonadism, chronic hepatitis, maldigestion syndrome, leucoderma, keratopathy and dysplasia of enamel may also occur. Even among siblings of the same family, the combination of clinical symptoms may not always be the same in terms of the affected organs and the progression of the disease. Various reports have described massive lymphocyte infiltrations into target organs as well as the detection of autoantibodies in the sera for target organs in patients with APECED. The frequency of APECED is reportedly high in certain genetically isolated groups such as Finnish (1/25,000), Iranian Jews (1/9,000) and Sardinians (1/14,000) groups [3], [4]. In 1997, autoimmune regulator (AIRE) was identified as the gene responsible for APECED on human chromosome 21 (q22.3) by using a positional cloning [5], [6]. Thereafter, various mutations of AIRE gene have been reported [7], [8]. The gene *AIRE* is about 12 kbp, consisting of 14 exons. AIRE protein is comprised of 545 amino acids and has an HSR (homogeneously staining region), two PHD (plant homeodomain) type zinc finger domains, three LXXLL motifs, a protein-rich domain, a SAND (Sp100, AIRE, NucP41/75, DEAP-1/suppression) domain, a nuclear transitional signal, and so on. HSR is thought to be a binding site when AIRE forms the dimer [9], [10], and it was reported that the SAND domain participates in the binding of DNA [11].

It has also been found that Aire KO mice produce autoantibodies for tissue-specific antigen in periphery and exhibit infiltration of lymphocytes into various tissues similarly to the findings in human APECED [12]–[17]. Although the affected organs including the liver, salivary glands, ovary and stomach in Aire KO autoimmune mice are not always the same as those target organs listed in human AIRE deficiency [12]–[14], these mice could serve as an excellent animal model for studying organ-specific autoimmune diseases.

Because Aire gene deficiency presents a vast autoimmune spectrum for target organs not only in humans but also in mice [12], [13], [17], [18], it is suggested that the clinical phenotype of Aire deficiency is further modulated under the influence of other factors, such as major histocompatibility complex (MHC) including human leukocyte antigen (HLA) [12], [19].

We conducted a histopathologic study, immunohistochemical staining and Western blot analysis, followed by a mass spectrometric analysis to delineate the cause of an inflammation in stomach and pancreas found in Aire KO mice with a BALB/cAnN background. This study presents evidence that Aire KO mice of BALB/cAnN background can serve as an excellent animal model for autoimmune gastro-pancreatitis.

## Materials and Methods

### Animals

Aire KO/C57BL/6 mice were obtained from Dr. L. Peltonen and Dr. L. Puhakka (Department of Medical Genetics, University of Helsinki). Aire KO/BALB/cAnNCrlCrlj (Aire KO/BALB/cAnN) mice were obtained after backcrossing for 13 generations onto BALB/cAnNCrlCrlj mice, which were purchased from Charles River. These mice were maintained under pathogen-free conditions and were handled in accordance with the Guidelines for Animal Experimentation of Kyushu University. The protocol was approved by the Committee on the Ethics of Animal Experiments of the University of Kyushu (Permit Number: A21-184-0, A23-021-0). All surgery was performed under sevoflurane anesthesia, and all efforts were made to minimize suffering. In immunohistochemical staining, we also used the spleen of the wild type LEW/CrlCrlj (Lewis)rat.

### Histopathology

Dissected tissues (pancreas, stomach and liver) were immersed in 10% formalin and fixed for over 24 h, and were then embedded in paraffin. Paraffin sections of three-micron thickness were stained using standard H&E staining protocols. Pancreases were obtained from mice and rats, and the frozen sections were first reacted with the sera from Aire KO or wild type mice, followed by DyLight 488™ conjugated affinity purified anti-mouse IgG (H&L) (Rockland Immunochemicals, Gilbertsville, PA). The evaluation of lymphocyte infiltration into pancreas was as follows, 0; none of lymphocyte infiltration, 1; lymphocyte infiltration only in perivascular area, 2; lymphocyte infiltration in the perivascular and the acinus tissue, 3; lymphocyte infiltration in the whole acinus tissue associated with breakdown of the acinus tissue due to fatty degeneration.

### Coomassie brilliant blue staining and Western blotting

Based on the immunohistochemical image suggesting the possible presence of autoantibody directed against pancreatic tissue antigens in Aire KO sera, we searched for the target antigen with Western blotting. As the primary antibodies, pooled sera were tested at the dilution of 1∶10, 1∶100, 1∶500, 1∶1000 and 1∶2000, and the clearest detection was obtained by 1∶500 dilution. We examined possible self-antigens in the pancreas, stomach, liver, thymus, kidney, submandibular gland, testis, epididymis, ovary, heart, brain, femoral muscle and pituitary gland. Each organ was homogenized in 20 mM HEPES buffer (pH 7.5) with protease inhibitor cocktail (Nacalai Tesque, Kyoto, Japan) and then sonicated. Homogenates were separated with centrifuge and the supernatants were subjected to 8% SDS-PAGE analysis. One gel was stained with Coomassie Brilliant Blue (CBB) and the bands on another gel were electrophoretically transferred to PVDF membrane (Hybond-P, Amersham Bioscience AB, Uppsala, Sweden). The membrane was blocked with Amersham ECL Advance Blocking Agent (GE Healthcare, Buckinghamshire, UK) and incubated at room temperature for 1 h with appropriate primary antibody in blocking buffer. Immunoreactivity was detected by sequential incubation with Anti-mouse IgG (H&L) HRP-linked secondary antibody (Cell Signaling Technology, Danvers, MA) and enzymatic chemiluminescence.

### MALDI-TOF/TOF MASS

The CBB-stained band corresponding to the possible autoantigen from Western blotting analysis was excised from the gel. The gel sample was sent to GENOMINE (Pohang, Korea) and analyzed by MALDI-TOF/TOF mass spectrometry for the identification of the protein.

### Protein expression and absorption test

The pdia2 protein expression vector, Ex-Mn 27257-B01, was purchased from GeneCopoeia (Rockville, MD). The vector was transformed to One Shot® BL21(DE3) (Life Technologies, Tokyo, Japan) and a colony of successful transformants was cultured in LB medium overnight with shaking. A portion of the culture was transferred to new LB medium and cultured until 0.4 of OD*600* was obtained. The expression of pdia2 was induced by addition of isopropylthio-beta-D-galactoside (IPTG) at 0.5 mM. After 2 h, the culture was centrifuged and the pellet was collected. This pellet was sonicated and centrifuged at 20,000×*g* for 15 min in PBS in the presence of protease inhibitor cocktail. This protein solution was mixed with each pool of the collected sera from 12-wk-old Aire KO or wild type mice and post-absorption serum obtained after an overnight incubation. Western blot analysis was done using both pre-absorption and post-absorption serum against pancreatic and gastric tissue samples.

### Analysis of antigen-specific T-cell response

Bone marrow-derived DC (BMDC) were prepared as described previously [20]. In brief, bone marrow cells were removed from the femurs and tibias of wild type mouse and cultured in RPMI 1640 medium containing L-glutamine (Life Technologies, Tokyo, Japan) supplemented with 10% heat inactivated fetal calf serum (FCS), 100 U ml^−1^ of penicillin, 100 µg ml^−1^ of streptomycin, 1∶50 of J558L-GMCSF supernatant (provided by Dr. Kobayashi, Oita University Graduate School of Medicine, Oita, Japan) as granulocyte macrophage colony-stimulating factor. On days 2 and 4, the culture medium was replaced with a fresh medium and cells were used on day 6 of culture. To prepare pdia2-pulsed APC, BMDC were treated with 10 µg ml^−1^ pdia2. On day 7, the cells were added with 1 µg ml^−1^ LPS (Sigma-Aldrich, St. Louis, MO). On day 8, the cells were treated with 50 µg ml^−1^ of mitomycin C (Sigma-Aldrich, St. Louis, MO) for 30 min at 37°C. CD4^+^ T cells (1×10^7^ cells ml^−1^) were obtained from spleens of wild and Aire KO mice by modified CD4^+^CD25^+^ Regulatory T cell Isolation Kit (Militenyi Biotec, Auburn, CA), and were stimulated with pdia2-pulsed and non-pulsed BMDC (1×10^4^ cells ml^−1^) for 72 h. Cell proliferation was measured by CellTiter 96® AQueous One Solution Cell Proliferation Assay (Promega corporation, Madison, WI). The plate was added with CellTiter 96® AQueous One Solution Reagent and incubated at 37°C for 4 h in a humidified, 5% CO_2_ atmosphere. The data were recorded as the absorbance at 490 nm corrected by a background and a reference wavelength of 655 nm.

### Gene expression analyses in thymus and pancreas

Total RNA from thymus and pancreas were prepared using ISOGEN (NIPPON GENE, Tokyo, Japan) and the concentration of total RNA was measured by NanoDrop ND-1000 (LMS, Tokyo, Japan). PCR was carried out on cDNA prepared using High-Capacity cDNA Reverse Transcription kit (Life Technologies, Tokyo, Japan) from 1 µg RNA. The following oligonucleotide pares were used: beta-actin: TGGAATCCTGTGGCATCCATGAAAC and TAAAACGCAGCTCAGTAACAGTCCG; C-reactive protein: CCATGGAGAAGCTACTCTG and CCCAAGATGATGCTTGC; Salivary protein 1: GGCTCTGAAACTCAGGCAGA and TGCAAACTCATCCACGTTGT; pdia2: AGAATGGAAACCGCACAAAC and AAGCCAAAGGTCATGTCCAG. PCR reactions were carried out in a final volume of 25 µl, using 0.25 U of *TaKaRa Ex Taq* (Takara Bio, Shiga, Japan) and 250 nM of each primer in 1×*Ex Taq* Buffer (Mg^2+^ free), 2 mM MgCl_2_ and 0.2 mM dNTP Mixture. Cycling conditions were 30 cycles of 98°C for 10 seconds, 55°C for 30 seconds and 72°C for 1 min.

### Statistical analysis

Data were analyzed using Mann-Whitney nonparametric test. For comparisons between two groups, the Student's *t*-test was used when the variances of the groups were judged to be equal by the *F*-test. Statistical significance was determined as *P*<0.05.

## Results

### Histopathology in Aire KO BALB/cAnN mice

**Figure 1:**
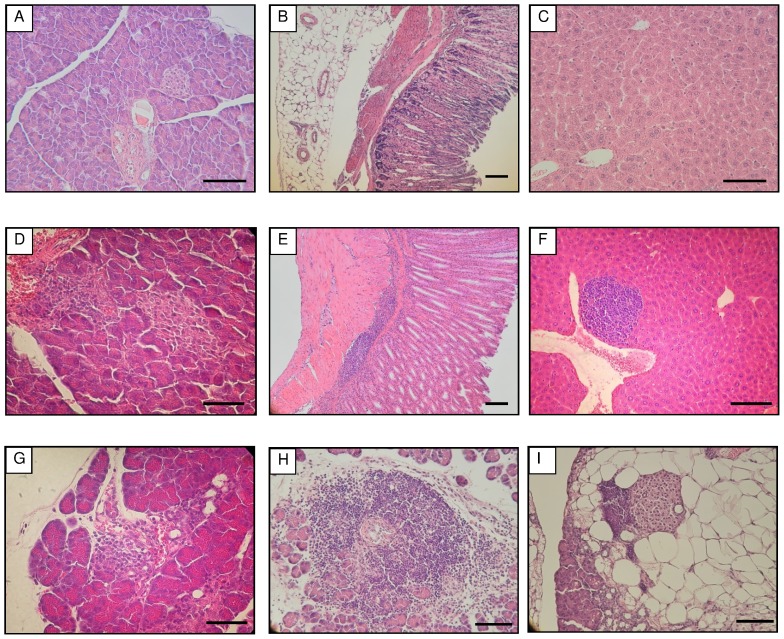
H&E-stained tissues of WT and Aire KO mice. A-C, pancreas, stomach and liver, respectively, from 12-wk-old WT mice. D-F, pancreas, stomach and liver, respectively, from 12-wk-old Aire KO mice. G, pancreas from 6-wk-old Aire KO mice. H and I, pancreas from 24-wk-old Aire KO mice. Each bar represents 100 µm.

H&E-stained tissue samples of stomach, liver and pancreas of Aire KO and wild type (WT) mice were examined under a microscope. In 12-wk-old WT mice, no pathological change was observed in the pancreas, stomach and liver (Figure 1A, B, C). In contrast, all three organs of Aire KO mice presented a remarkable infiltration of lymphocytes (Figure 1D, E, F). The infiltration of lymphocytes in the pancreas was mainly detected around blood vessels and moreover into acini (Figure 1D). In the stomach, cell infiltration was found from the surrounding areas of the blood vessels to the lamina muscularis mucosae and tunica propria (Figure 1E). In the liver, the infiltration was observed at the edge of the central vein and often expanded to hepatocytes (Figure 1F). Strikingly, the pancreas at 6-wk-old Aire KO mice was affected by infiltration of lymphocytes from the surroundings of blood vessels to acini (Figure 1G), and the significant progression of lymphocytic infiltrations (Figure 1H) associated with extensive fatty change of the acinar cells (Figure 1I) was observed in 24-wk-old Aire KO mice. Thus, the main target organ of inflammatory reaction was directed against the pancreas.

### Immunohistochemical study and histological scores in the pancreas

**Figure 2:**
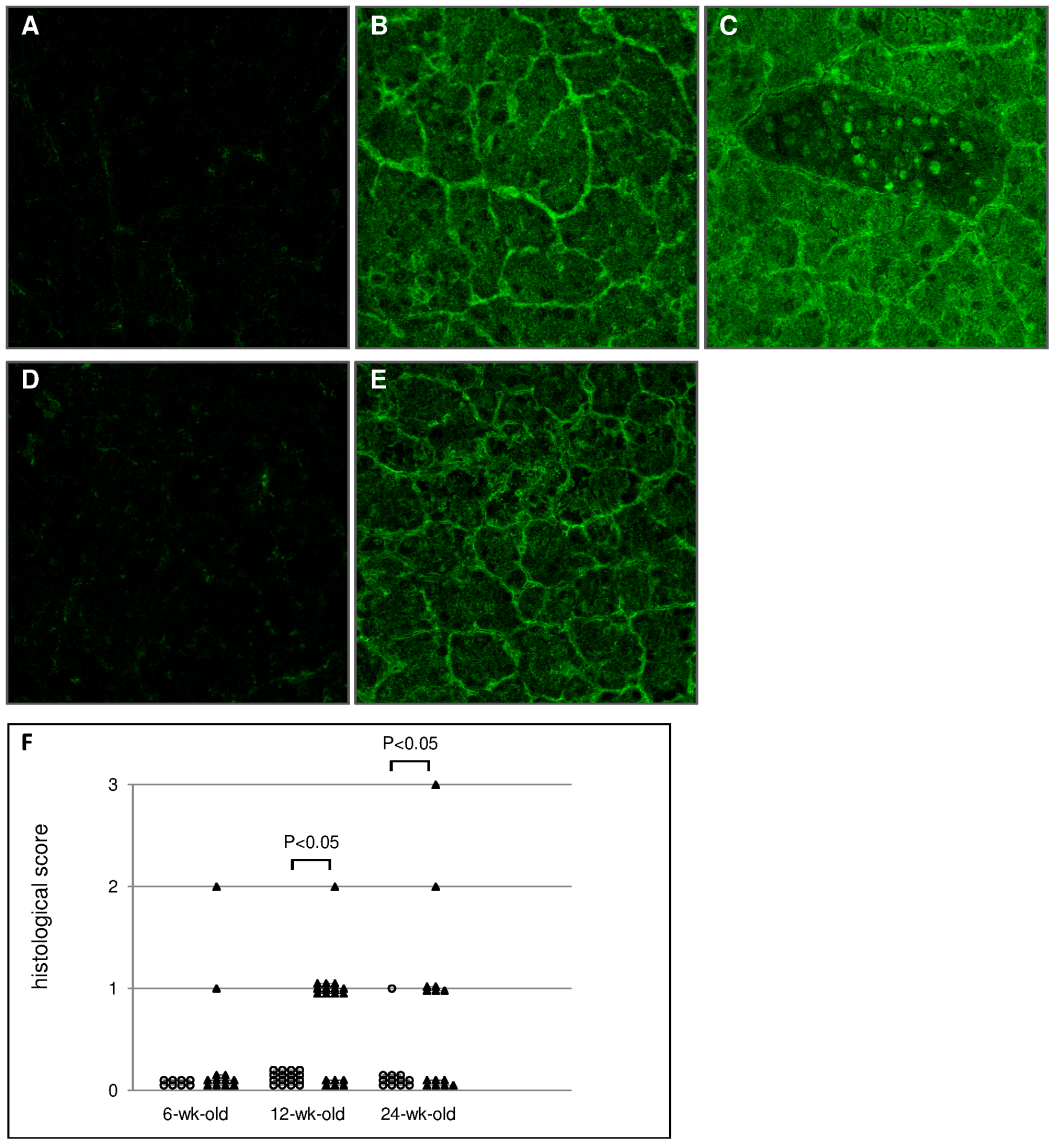
Immunohistochemistry and histological scores. Immunohistochemistry of frozen sections in mouse pancreas (A, B, C) and in rat pancreas (D, E). A and D were reacted by primary antibody of WT mice pooled sera. B, C and E were reacted by primary antibody of Aire KO mice pooled sera. B, C and E show strong fluorescence in the whole tissue. F, histological scores of Aire WT (open circles) and Aire KO (closed triangles) are that the evaluation of lymphocyte infiltration into pancreas was as follows, 0; none of lymphocyte infiltration, 1; lymphocyte infiltration only in perivascular area, 2; lymphocyte infiltration in the perivascular and the acinus tissue, 3; lymphocyte infiltration in the whole acinus tissue associated with breakdown of the acinus tissue due to fatty degeneration.

Based on the histopathological study, the infiltration of lymphocytes into pancreas in Aire KO BALB/cAnN mice seemed to be caused by an autoimmune reaction. We examined frozen sections by immunohistochemistry with the primary antibody of Aire KO or WT mouse sera (Figure 2A-E). Because the secondary antibody was anti-mouse IgG (H&L), positive staining of the tissue might be a non-specific reaction due to circulating serum IgG. To exclude such a possible non-specific reaction, we also examined WT rat pancreas as well as mouse pancreas as the target organ to be studied (Figure 2D, E). In both mouse and rat, possible non-specific fluorescence was minimal when pooled sera of WT mice were used as the primary antibody (Figure 2A, D). By contrast, when the pooled sera from Aire KO mice were used, a significantly strong and specific fluorescence was detected (Figure 2B, C, E). Thus, the presence of non-species-specific autoantibody against the pancreatic tissue in Aire KO mouse serum was confirmed. The whole pancreas, especially pancreatic acinar cells, showed a strong fluorescence, while pancreatic islets showed only weak fluorescence. Because only a part of the nucleus in pancreatic acinar cells showed a low level and partial fluorescence, it was suggested that the target antigen of autoantibody in Aire KO mouse serum was mainly in the cytoplasm of the pancreatic acinar cells. The rate of lymphocyte infiltration in pancreas was 7/14 (50.0%) in 24-wk-old Aire KO mice, 12/18 (66.7%) in 12-wk-old Aire KO mice and 2/12 (16.7%) in 6-wk-old Aire KO mice. Tissue sections were scored on a grading system from 0 to 3 (Figure 2F).

### Search for target antigen of autoantibody in Aire KO mice by Western blotting

**Figure 3:**
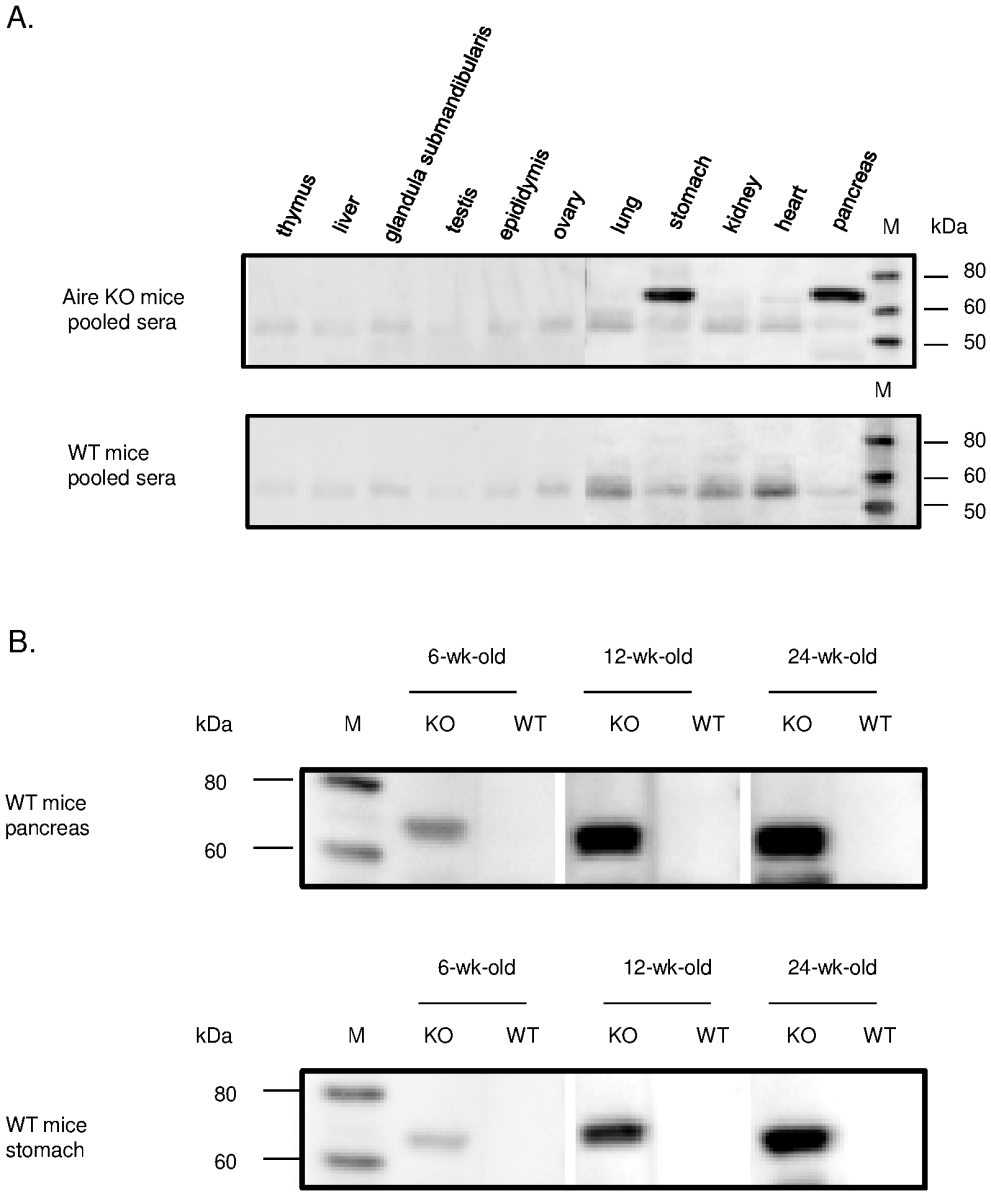
The search for target antigen in Aire KO mice serum with Western blotting. A. With the sample for the other organ tissue protein homogenates(thymus, liver, submandibular gland, testis, epididymis, ovary, lung, stomach, kidney, heart and pancreas), the search for autoantibody for tissue antigen. Upper shows Aire KO mice pooled sera for the primary antibody, lower shows WT mice pooled sera for the primary antibody. B. Western blotting for pancreatic and gastric protein with KO and WT mice pooled sera. M: marker.

Subsequently, we also examined the reaction of autoantibody in the pooled sera from Aire KO mice for several organs (thymus, liver, submandibular gland, testis, epididymis, ovary, lung, stomach, kidney, heart, and pancreas) (Figure 3A). Positive reaction was observed with tissue antigen of approximately 70 kDa in gastric and pancreatic tissues. No band of this size was detected in any of the organs other than the stomach and pancreas. Therefore, we also examined the dependence of the age for the presence of autoantibody directed against pancreatic and gastric tissue antigen by using pooled sera (Figure 3B). The number of mice was: 6-wk-old Aire KO (n = 12), 12-wk-old Aire KO (n = 19), 24-wk-old Aire KO (n = 16), 6-wk-old WT (n = 9), 12-wk-old WT or hetero (n = 21) and 24-wk-old WT (n = 12). Individual Aire KO mouse serum was reacted to the pancreatic and gastric tissue antigen of 70 kDa in size. The rates of autoantibody detection in Aire KO mice serum against pancreas were 12/16 (75.0%) in 24-wk-old Aire KO, 4/5 (80.0%) in males and 8/11 (72.7%) in females. Similarly, in 12-wk-old Aire KO mice, the total rate was 10/19 (52.6%), being 5/9 (55.6%) in males and 5/10 (50.0%) in females. In 6-wk-old Aire KO, the total rate was 1/12 (8.0%) and that in males was 1/9 (16.7%). (Figure S1A-C).

### Identification of target antigen by MALDI-TOF/TOF Mass analysis

**Figure 4:**
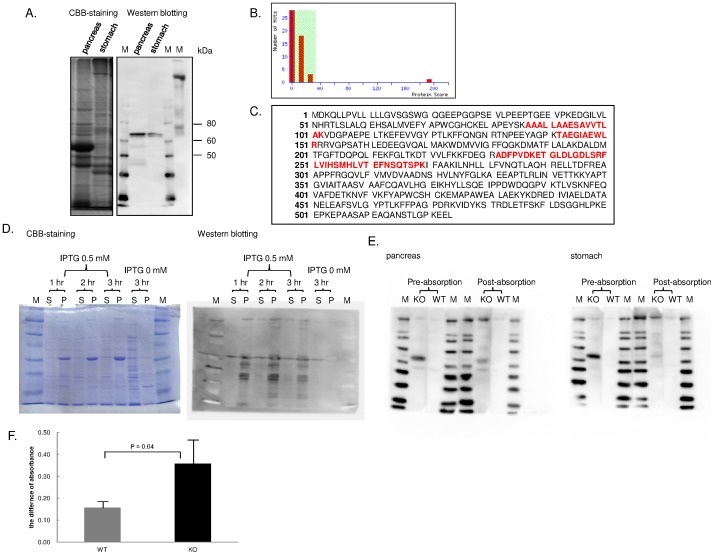
Identification of the target antigen by MALDI-TOF/TOF Mass analysis. A. CBB-staining and Western blotting analysis with pancreas tissue sample. Band corresponding to Western blotting was cut from CBB-stained gel and analyzed by MALDI-TOF/TOF Mass. M: marker. B. Number of hits of candidate protein by MALDI-TOF/TOF Mass analysis and its significance score. Because the left part was lower than the threshold of protein score, that part had a lower confidence in candidate protein. Only Pdia2 had the high score, to be a sufficient candidate molecule as a target antigen. C. Sequence of Pdia2 identified by MALDI-TOF/TOF analysis and other four peptide sequences suggested from the analysis data. Sequence coverage was 12%. D. Pdia2 protein expression in BL21. M: marker, S: supernatant, P: pellet. E. Absorption test for WT mouse pancreas and stomach in 12-wk-old Aire KO or WT mice pooled sera. M: marker. F. The difference of the absorbance in WT or KO mouse CD4+ T cells stimulated with pdia2-pulsed BMDC. Each graph represents the mean ± SD of 3 replicates.

To identify the molecule of target antigen as detected by Western blotting, the pancreas homogenate was separated by electrophoresis on 8.0% SDS-PAGE. One-half was stained with CBB and the other was transferred to PVDF membrane and subjected to Western blotting analysis. The band corresponding to the possible autoantigen was excised out and examined by MALDI-TOF/TOF MS analysis (Figure 4A). As the result, 5 different proteins were proposed as the candidate antigen, including protein disulfide isomerase-associated 2 (Pdia2), alpha amylase, angiotensin II receptor-interacting protein, LDL receptor member LR3 and OL-protocadherin. Each candidate protein had the following significance score: Pdia2, 193; alpha amylase, 24; angiotensin II receptor-interacting protein, 20; LDL receptor member LR3, 20; OL-protocadherin, 18. Because the cut-off value of the significance score was 33 in this assay system, the protein in the excised gel was identified to be Pdia2 (Figure 4B). The sequence coverage between the peptide fragment and Pdia2 protein sequence was 12% as shown in red for the corresponding sequences (Figure 4C). By using Ex-Mn 27257-B01 vector system, we attempted the expression of rPdia2 in *E. coli* in several conditions (Figure 4D). To confirm that the target antigen detected as 70 kDa protein is Pdia2, we finally conducted an absorption test. The expressed protein solution and each pooled sera from 12-wk-old Aire KO or WT mice were mixed overnight, and post-absorption serum was prepared. Absorption test was performed by Western blotting analysis using pre- and post-absorption serum against pancreatic and gastric tissue samples. In both pancreatic and gastric tissue sample, an obvious decrease in Western blotting was observed when post-absorption serum was used, thus confirming the specificity of autoantibody in the Aire KO mouse serum being directed against Pdia2 (Figure 4E). To clarify whether Pdia2 is recognized by reactive T cells in Aire KO mice, we carried out lymphocyte proliferation assay on CD4^+^ T cell in Aire KO and WT mice to check antigen-specific CD4^+^ T cell response. CD4^+^ T cells in Aire KO mouse exhibited a significantly higher proliferative response than those in WT mouse (Figure 4F). Moreover, to examine the ectopic expression of peripheral tissue-restricted antigens, we performed reverse-transcription PCR (RT-PCR) analyses on cDNA from thymus of Aire KO and WT mice. Although salivary protein 1 and pdia2 were absent or reduced in Aire KO mouse, beta-actin and C-reactive protein were still expressed (Figure S2).

## Discussion

It is well known that Aire deficiency leads to an autoimmne reaction directed against a wide spectrum of various organs, and the clinical phenotypes differ depending on the genetic background not only in humans but also in mice. Aire KO mice of SJL/J and C57BL/6J backgrounds are reported to exhibit a massive infiltration of lymphocytes associated with the autoantibodites to various organs [13]. Aire KO mouse of NOD background is known to develop destruction in pancreatic acini due to autoimmune reaction [15]. The phenotype of BALB/cJ background Aire KO mouse has been most extensively analyzed in detail. Interestingly, it is reported that inflammation exists in the cornea (60%), salivary gland (20%), stomach (100%), ovary (100%), lung (50%), liver (40%) and prostate (100%). In addition, the detection of autoantibody was reported only for the eye (33%) and stomach (100%). In spite of the autoimmune response to a variety of organs, lymphocyte infiltration in the pancreas and the autoantibody production for pancreatic tissue have not so far been reported in BALB/cJ mice [17].

In contrast, as clearly shown in this study, an obvious infiltration of lymphocytes into pancreas and stomach was observed in BALB/cAnN background. Our study confirmed that the autoimmune reaction observed in pancreas is due to the autoantibody production against Pdia2 (protein disulfide isomerase A2), also known under the name of pancreas-specific protein disulfide isomerase (PDIp). The production of anti-Pdia2 autoantibody correlated well with the progression of autoimmunity as revealed by the infiltration of lymphocytes in the pancreas, stomach, liver and other internal organs in accordance with aging (Figure 1).

No difference was observed in the detection rate of autoantibody between male and female groups (Figure S1A-C). However, the percentage of autoantibody detection increased from 8.0% in 6-wk-old Aire KO mice to 75.0% in the 24-wk-old group. These data indicated that the progression of autoimmunity to specific organs is highly dependent on the age in Aire KO BALB/cAnN mice. This fact is consistent with human organ-specific autoimmune diseases.

The novel finding obtained in this study using in BALB/cAnN is different from previous report [17]. The difference of the autoimmune disease particularly regarding the affected organs between BALB/cJ and BALB/cAnN may be caused by the difference in breeding environment including the microbiota or other unidentifiable mechanisms including minor genetic background difference. To investigate the cause of the appearance of the autoantibody, the expression of several genes, such as C-reactive protein and Salivary protein 1, was examined in thymus and pancreas in Aire WT or KO mouse by RT-PCR. The result on beta-actin, C-reactive protein and salivary protein 1 was the same as the previous report [14]. Although the significant expression of Pdia2 was observed in WT pancreas, it was scarcely detected in WT nor KO mice (Figure S2). Thus, some mechanism other than thymus-dependent central tolerance, such as peripheral autoimmune regulatory mechanisms dependent on Aire, may be involved in the regulation of auto-reactivity to Pdia2 in BALB/cAnN mice.

In the present study, we identified Pdia2 protein as the main autoantigen involved in the autoimmune response observed in Aire KO mice of BALB/cAnN background. Pdia2 protein was presumed to be a specific protein in the pancreas, especially in pancreatic acinar cells [21]. Later on, this protein has been found to exist also in the mouse organs including stomach, vermiform appendix, ileum, adrenal gland, epididymis and testis, in addition to the pancreatic acinar cells [22], [23]. Our study showed that the autoantibody Aire KO BALB/cAnN mice reacted to only pancreatic and gastric tissues, because Pdia2 protein was more highly expressed in the digestive organs than in the other organs [23]. To date, the significance of the autoantibodies of human autoimmune pancreatitis has already been reported, such as antinuclear antibody, rheumatoid factor, anti-carbonic anhydrase II antibody and anti-lactoferrin antibody. It was also reported that the peptide homologous to UBR2 (ubiquitin-protein ligase E3 component n-recognin 2), which is an enzyme highly expressed in pancreatic acinar cells, was also the target antigen of autoantibody detected in the serum of patients suffering from autoimmune pancreatitis [24].

Although many kinds of tissue antigens could be the target antigens in human autoimmune pancreatitis, the anti-Pdia2 antibody identified in this study present the evidence that the autoantibody could serve as an important autoantibody in autoimmune gastritis and/or pancreatitis, at least in mice. Thus, Aire KO BALB/cAnN mice would be an excellent animal model to reveal the developmental mechanism of autoimmune gastritis and/or pancreatitis. Further studies are required to disclose whether autoantibody to Pdia2 may also play a role in human gastritis and/or pancreatitis, or other organ-specific autoimmune diseases.

## Supporting Information

Figure S1 **The search for pancreatic protein in individual Aire KO mouse serum with Western blotting.** A-C shows the autoreactivity by individual serum from 6-wk-old Aire KO mice, 12-wk-old Aire KO mice and 24-wk-old Aire KO mice, respectively. Upper numbers indicate the identification numbers of mice. M: marker.
(ZIP)

Figure S2 **Gene expression analyses in thymus and pancreas.** To investigate the cause of the appearance of the autoantibody, the expression of several genes, such as beta-actin, C-reactive protein, Salivary protein 1 and pdia2, was examined in thymus and pancreas in Aire WT or KO mouse by RT-PCR.
(ZIP)

## References

[1] Takahama Y (2006) Journey through the thymus: stromal guides for T-cell development and selection. Nat Rev Immunol 6: 127–135. 16491137 10.1038/nri1781

[2] Anderson G, Lane PJL, Jenkinson EJ (2007) Generating intrathymic microenvironments to establish T-cell tolerance. Nat Rev Immunol7: 954–963. 10.1038/nri2187 17992179

[3] Peterson P, Org T, Rebane A (2008) Transcriptional regulation by AIRE: molecular mechanisms of central tolerance. Nat Rev Immunol 8: 948–957. 19008896 10.1038/nri2450 PMC2785478

[4] Peterson P, Peltonen L (2005) Autoimmune polyendocrinopathysymdrome type 1 (APS1) and AIRE gene: New views on molecular basis of autoimmunity. J Autoimmun 25: 49–55. 16290093 10.1016/j.jaut.2005.09.022

[5] Nagamine K, Pelerson P, Scott HS, Kudoh J, Minoshima S, et al (1997) Positional cloning of the APECED gene. Nat Genet 17: 393–398. 9398839 10.1038/ng1297-393

[6] The Finnish-German APECED Consortium (1997) An autoimmune disease, APECED, caused by mutations in a novel gene featuring two PHD-type zinc-finger domains. Nat Genet 17: 399–403. 9398840 10.1038/ng1297-399

[7] Scott HS, Heino M, Peterson P, Mittaz L, Lalioti MD, et al (1998) Common mutations in autoimmune polyendocrinophathy-candidiasis-ectodermal dystrophy patients of different origins. Mol Endocrinol 12: 1112–1119. 9717837 10.1210/mend.12.8.0143

[8] Pearce SHS, Cheetham T, Imrie H, Vaidya B, Barnes ND, et al (1998) Common and recurrent 13-bp deletion in the autoimmune regulator gene in British kindreds with autoimmune polyendocrinopathy Type 1. Am J Hum Genet 63: 1675–1684. 9837820 10.1086/302145 PMC1377639

[9] Sternsdorf T, Jensen K, Reich B, Will H (1999) The nuclear dot protein Sp100, characterization of domains necessary for dimerization, subcellular localization, and modification by small ubiquitin-like modifiers. J Biol Chem 274: 12555–12566. 10212234 10.1074/jbc.274.18.12555

[10] Pitkänen J, Doucas V, Sternsdorf T, Nakajima T, Aratani S, et al (2000) The autoimmune regulator protein has transcriptional transactivating properties and interacts with the common coactivator CREB-binding protein. J Biol Chem 275: 16802–16809. 10748110 10.1074/jbc.M908944199

[11] Gibson TJ, Ramu C, Gemünd C, Aasland R (1998) The APECED polyglandular autoimmune syndrome protein, AIRE-1, contains the SAND domain and is probably a transcription factor. Trends Biochem Sci 23: 242–244. 9697411 10.1016/s0968-0004(98)01231-6

[12] Kuroda N, Mitani T, Takeda N, Ishimaru N, Arakaki R, et al (2005) Development of autoimmunity against transcriptionally unrepressed target antigen in the thymus of Aire-deficient mice. J Immunol 174: 1862–1870. 15699112 10.4049/jimmunol.174.4.1862

[13] Ramsey C, Winqvist O, Puhakka L, Halonen M, Moro A, et al (2002) Aire deficient mice develop multiple features of APECED phenotype and show altered immune response. Hum Mol Genet 11: 397–409. 11854172 10.1093/hmg/11.4.397

[14] Anderson MS, Venanzi ES, Klein L, Chen Z, Berzins SP, et al (2002) Projection of an immunological self shadow within the thymus by the Aire protein. Science 298: 1395–1401. 12376594 10.1126/science.1075958

[15] Niki S, Oshikawa K, Mouri Y, Hirota F, Mastushima A, et al (2006) Alteration of intra-pancreatic target-organ specificity by abrogation of Aire in NOD mice. J Clin Invest 116: 1292–1301. 16628255 10.1172/JCI26971 PMC1440703

[16] Mathis D, Benoist C (2007) A decade of Aire. Nat Rev Immunol 7: 645–650. 17641664 10.1038/nri2136

[17] Jiang W, Anderson MS, Bronson R, Mathis D, Benoist C (2005) Modifier loci condition autoimmunity provoked by Aire deficiency. J Exp Med 202: 805–805. 16172259 10.1084/jem.20050693 PMC2212943

[18] Venanzi ES, Melamed R, Mathis D, Benoist C (2008) The variable immunological self: Geneic variation and nongenetic noise in Aire-regulated transcription. Proc Natl Acad Sci USA 105: 15860–15865. 18838677 10.1073/pnas.0808070105 PMC2572942

[19] Kogawa K, Kudoh J, Nagafuchi S, Ohga S, Katsuta H, et al (2002) Distinct clinical phenotype and immunoreactivity in Japanese siblings with autoimmune polyglandular syndrome type 1 (APS-1) associated with compound heterozygous novel AIRE gene mutation. Clin Immunol 103: 277–283. 12173302 10.1006/clim.2002.5208

[20] Inaba K, Inaba M, Romani N, Aya H, Deguchi M, et al (1992) Generation of large numbers of dendritic cells from mouse bone marrow cultures supplemented with granulocyte/macrophage colony-stimulating factor. J Exp Med 176: 0693–0702. 10.1084/jem.176.6.1693 PMC2119469 1460426

[21] Desilva MG, Lu J, Donadel G, Modi WS, Xie H, et al (1996) Characterization and chromosomal localization of a new protein disulfide isomerase, PDIp, highly expressed in human pancreas. DNA Cell Biol 15: 9–16. 8561901 10.1089/dna.1996.15.9

[22] Horiuchi A, Kawa S, Akamatsu T, Aoki Y, Mukawa K, et al (1997) Characteristic pancreatic duct appearance in autoimmune chronic pancreatitis: a case report and review of the Japanese literature. Am J Gastroenterol 93: 260–263. 10.1111/j.1572-0241.1998.00260.x 9468255

[23] Fu XM, Dai X, Ding J, Zhu BT (2009) Pancreas-specific protein disulfide isomerae has a cell type-specific expression in various mouse tissues and is absent in human pancreatic adenocarcinoma cells: implications for its function. J Mol Histol 40: 189–199. 19821078 10.1007/s10735-009-9230-5

[24] Frulloni L, Lunardi C, Simone R, Dolcino M, Scattolini C, et al (2009) Identification of a novel antibody associated with autoimmune pancreatitis. N Engl J Med 361: 2135–2142. 19940298 10.1056/NEJMoa0903068

