# Supplementary figures and images for: Autoimmune Gastro-Pancreatitis with Anti-Protein Disulfide Isomerase-Associated 2 Autoantibody in Aire-Deficient BALB/cAnN Mice
Source: PLoS One. 2013 Aug 26;8(8):e73862. doi: 10.1371/journal.pone.0073862 (PMC3753263; doi:10.1371/journal.pone.0073862)

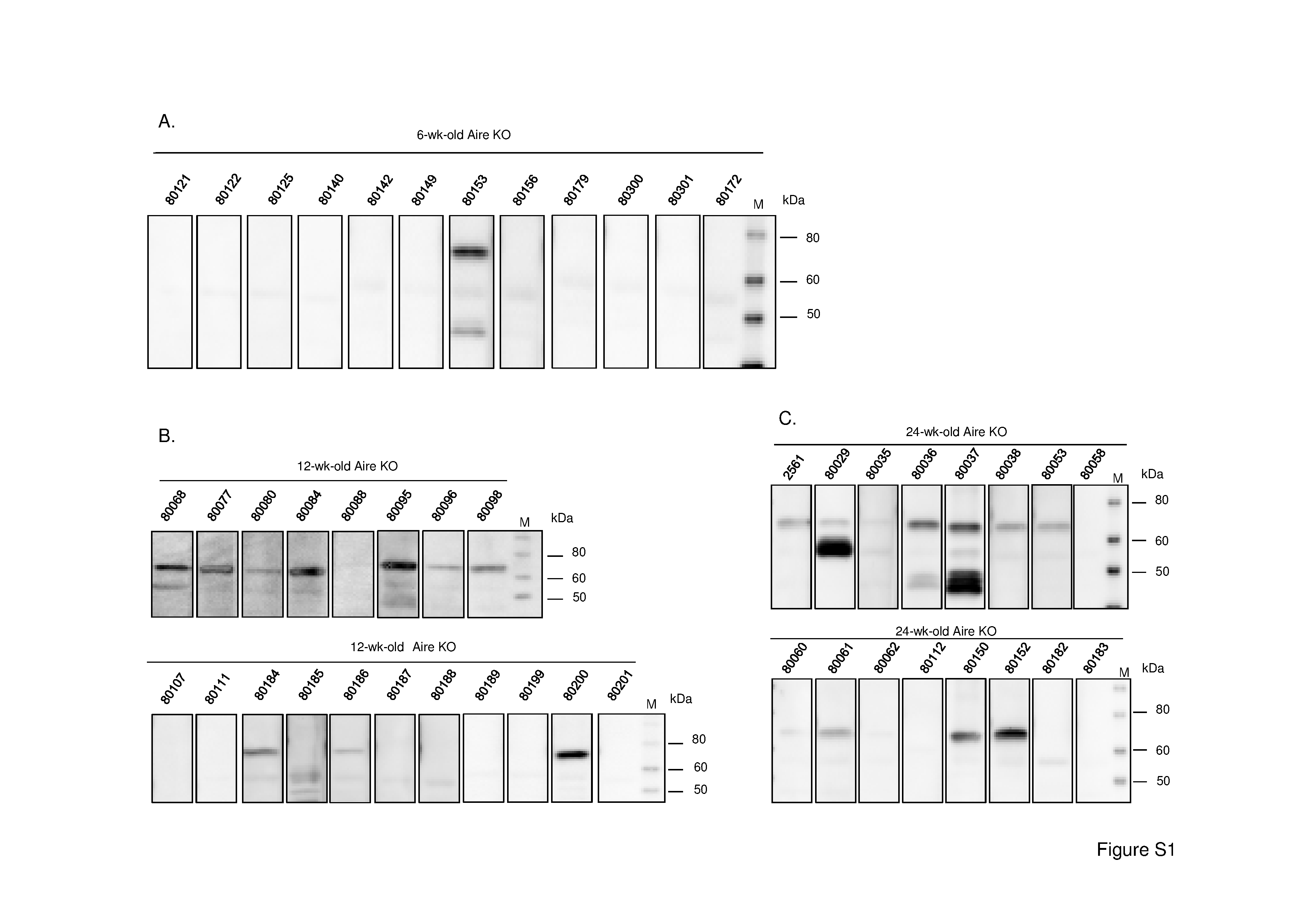

Supplement: Figure S1 — The search for pancreatic protein in individual Aire KO mouse serum with Western blotting. A-C shows the autoreactivity by individual serum from 6-wk-old Aire KO mice, 12-wk-old Aire KO mice and 24-wk-old Aire KO mice, respectively. Upper numbers indicate the identification numbers of mice. M: marker. (ZIP) [file pone.0073862.s001.zip › figure S1.tif]

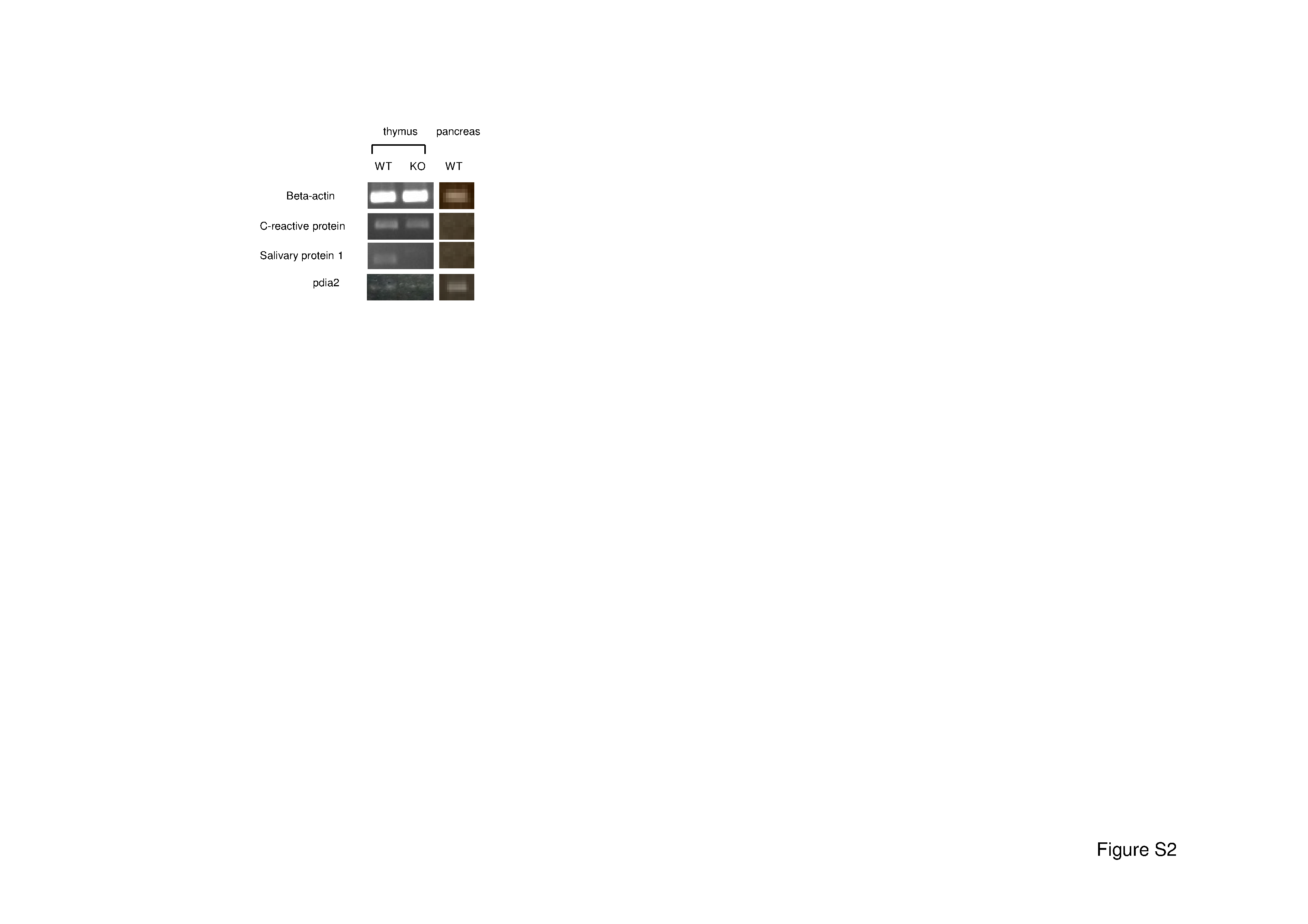

Supplement: Figure S2 — Gene expression analyses in thymus and pancreas. To investigate the cause of the appearance of the autoantibody, the expression of several genes, such as beta-actin, C-reactive protein, Salivary protein 1 and pdia2, was examined in thymus and pancreas in Aire WT or KO mouse by RT-PCR. (ZIP) [file pone.0073862.s002.zip › figure S2.tif]
